# Risk factors and prediction model for inadvertent intraoperative hypothermia in patients undergoing robotic surgery: a retrospective analysis

**DOI:** 10.1038/s41598-023-30819-1

**Published:** 2023-03-06

**Authors:** Zhouting Hu, Wangyu Li, Chen Liang, Kai Li

**Affiliations:** 1 Department of Anesthesiology, China-Japan Union Hospital, Jilin University, 126th Xiantai Avenue, Changchun, 130021 Jilin People’s Republic of China; 2 Vilcek Institute of Graduate Biomedical Sciences, New York University, New York, NY USA; 3 Outcomes Research Consortium, Cleveland, OH USA

**Keywords:** Health care, Medical research, Risk factors

## Abstract

This study explored the risk factors and established a prediction model for intraoperative hypothermia (IOH) in patients undergoing robotic surgery. We conducted a retrospective survey of patients undergoing elective robotic surgery at the China-Japan Union Hospital of Jilin University during June 2020–October 2021 using institutional medical records. Intraoperative core temperatures and potential influencing factors were collected, and regression analyses were used to assess the risk factors for IOH and establish a prediction model for the incidence of IOH. Overall, 833 patients who underwent robotic surgery were included in the final analysis; IOH was observed in 344 patients (incidence, 0.41; 95% confidence interval [CI] 0.38–0.45). A higher body mass index (BMI) and baseline core temperature were protective factors for IOH. A final prediction model for IOH was developed based on the determining factors with an area under the receiver operating characteristic curve of 0.85 under fivefold cross validation (95% CI 0.83–0.88). Accordingly, a lower BMI and baseline core temperature, thoracic surgeries, morning surgeries, and surgeries with longer durations were risk factors for IOH during robotic surgeries. Our prediction model has an excellent discrimination ability for predicting IOH in robotic surgeries.

## Introduction

Inadvertent intraoperative hypothermia (IOH), which is defined as a core temperature of < 36 °C, is recognized as a common adverse event among patients undergoing surgery under general anesthesia^1^. It has been shown that IOH is related to numerous complications, including surgical site infection, thrombosis, disturbed drug metabolism, and delayed emergence^2–5^. In addition, researchers have found that mild IOH increases blood loss, whereas aggressive thermal management reduces blood transfusion^6–8^. Recent studies have reported several factors associated with the incidence of IOH, including age > 65 years, low body weight or poor nutritional status, general anesthesia combined with high-level neuraxial anesthesia for its corresponding sympatholytic effect, intraoperative infusion with large volumes of unwarmed solutions, transfusion of cold red blood cells, and duration of anesthesia > 2 h^1,9^.

Compared with ordinary laparoscopic and thoracoscopic operations, robotic surgery is commonly performed with a longer surgical duration and unwarmed carbon dioxide for artificial pneumoperitoneum^10,11^. A wider surgical area in which more trocars can be inserted results in a broader body exposure and less skin surface available for active warming. These factors might lead to different incidences and risk factors for IOH in robotic surgery compared with routine surgery.

Therefore, our retrospective analysis aimed to investigate the incidence of IOH, examine the risk factors, and establish a prediction model for IOH, specifically for robotic surgery.

## Results

**Figure 1:**
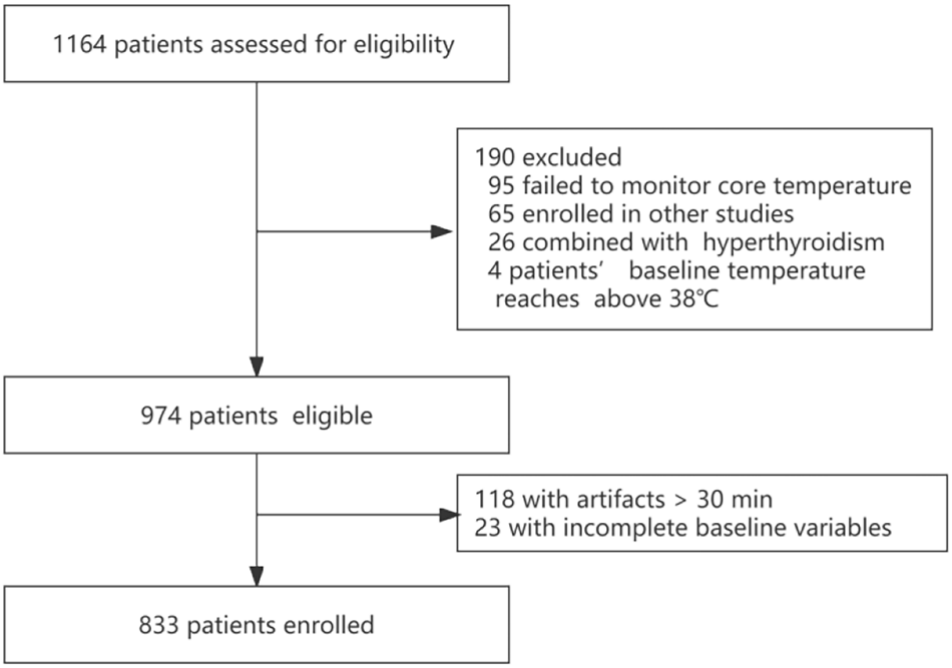
Flowchart.

Overall, 1164 patients were screened for eligibility, among whom 190 were excluded for the reasons explained in Fig. 1. Another 118 patients whose temperature artifacts lasted more than 30 min were excluded from the study, and 856 patients were considered for the analysis. Furthermore, after excluding 23 patients with incomplete baseline variables, 833 were included in the final analysis.

**Figure 2:**
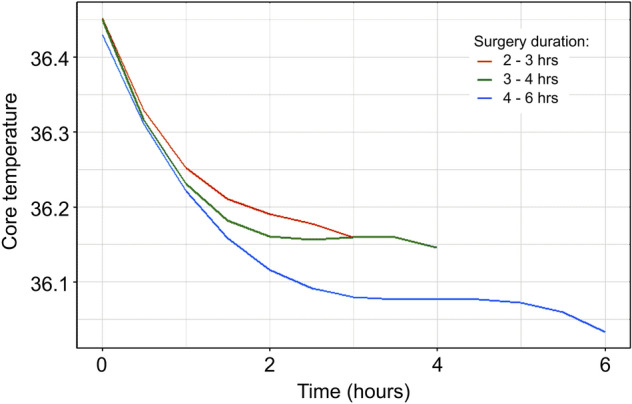
Trend of core temperature using B-spline. The core temperature trend was generated using linear mixed models with random intercepts for each patient. The nonlinearity of the time effect was incorporated using B-splines. Baseline core temperatures were also adjusted in the model. Models were constructed separately for surgical durations of 2–3 h, 3–4 h, and 4–6 h.

We provided boxplots for core temperatures according to surgical duration to examine the core temperature trends, as shown in Supplementary Fig. S1. From the boxplot, the core temperatures of the patients tended to decrease during the first 2 h and subsequently remained relatively stable. In addition, similar patterns were observed when we modeled the core temperature trend using spline terms (Fig. 2). And we provieded descriptive data on blood loss, transfusion, blood transfusion and irrigation in supplementary Table 1.

Among the 833 patients included in the analysis, we observed IOH in 344 patients, with an incidence rate of 0.41 (95% confidence interval [CI] 0.38–0.45). The incidence rates in abdominal, thoracic, and thyroid surgeries were 0.41 (95% CI 0.37–0.45), 0.55 (95% CI 0.46–0.64), and 0.32 (95% CI 0.24–0.41), respectively. Older patients, male, and those with a lower body mass index (BMI) and baseline core temperature, were more likely to experience IOH. In addition, IOH was more likely to occur during morning surgeries and in those with longer durations. Intraoperatively, patients with IOH experienced more blood loss and received more fluid and blood transfusions. The patient profiles are presented in Table 1.

**Table 1:** Patient demographics and anesthesia/surgery data (N = 833).

	IOH (N = 344)	No IOH (N = 489)	*P*-value
Age	54 ± 15	48 ± 15	< 0.01
Female	182 (53)	313 (64)	< 0.01
BMI, mean ± SD	26 ± 6	27 ± 7	< 0.01
Diabetes	40 (12)	46 (9)	0.36
ASA			0.33
1	6 (2)	5 (11)	
2	252 (73)	384 (79)	
3	85 (25)	99 (20)	
4	1 (< 1)	1 (< 1)	
Anesthesia induction time			< 0.01
Morning	195 (57)	190 (39)	
Afternoon	102 (30)	198 (40)	
Evening	47 (14)	101 (21)	
Surgical site (%)			< 0.01
Abdominal	239 (69)	351 (72)	
Thoracic	65 (19)	53 (11)	
Thyroid	40 (12)	85 (17)	
Surgical duration (min)	224 ± 90	200 ± 72	< 0.01
Baseline core temperature	36.1 ± 0.3	36.6 ± 0.3	< 0.01
Room temperature	23.7 ± 1.1	23.8 ± 1.1	0.17

Data are summarized as the mean ± SD or N (%).

IOH, inadvertent intraoperative hypothermia; BMI, body mass index; ASA, American Society of Anesthesiologists; SD, standard deviation.

Using all baseline variables as candidate predictors, BMI, baseline core temperature, surgery time, surgical site, and duration of anesthesia were retained in the model. Notably, in the multivariable model, a higher BMI and baseline core temperature were protective factors that prevented IOH. Each 5 kg/m^2^ increase in BMI was associated with a 0.96 (95% CI 0.95–0.99; *P* = 0.002) lower odds for IOH, whereas each 1 °C increase in baseline core temperature was associated with a 0.52 (95% CI 0.48–0.55; *P* < 0.001) lower odds. Patients who underwent thoracic surgery were more likely to develop hypothermia than those who underwent thyroid surgery (odds ratio [OR], 1.24; 95% CI 1.12–1.38; *P* < 0.001). In contrast, there was no significant difference between those who underwent thyroid and abdominal surgeries (OR, 1.06; 95% CI 0.98–1.15; *P* = 0.16) (Table 2). Patients who underwent anesthesia induction in the morning had 1.08 (95% CI 1.01–1.15; *P* = 0.02) higher odds for IOH than those in the afternoon. However, no significant difference was observed between those induced in the evening and afternoon (OR, 0.98, 95% CI 0.90–1.06; *P* = 0.61). In addition, each 1 h longer surgical duration was associated with a 1.05 (95% CI 1.02–1.07; *P* < 0.001) higher odds for IOH. Although an exact surgical duration is not available before surgery, an approximate duration can be forecast and used in the prediction model. The model specifications are presented in Table 2.

**Table 2:** Risk factors associated with intraoperative hypothermia.

	Adjusted OR (95% CI)	*P*-value
BMI (per 5 kg/m^2^)	0.96 (0.95–0.99)	0.002
Baseline core temperature (per 1 °C)	0.52 (0.48–0.55)	< 0.001
Anesthesia induction time		
Morning	1.08 (1.01–1.15)	0.02
Afternoon	Reference	0.61
Evening	0.98 (0.90–1.06)	
Surgical site		
Thyroid	Reference	
Abdominal	1.06 (0.98–1.15)	0.16
Thoracic	1.24 (1.12–1.38)	< 0.001
Surgical duration (per 1 h)	1.05 (1.02–1.07)	< 0.001

OR, odds ratio; CI, confidence interval; BMI, body mass index.

**Figure 3:**
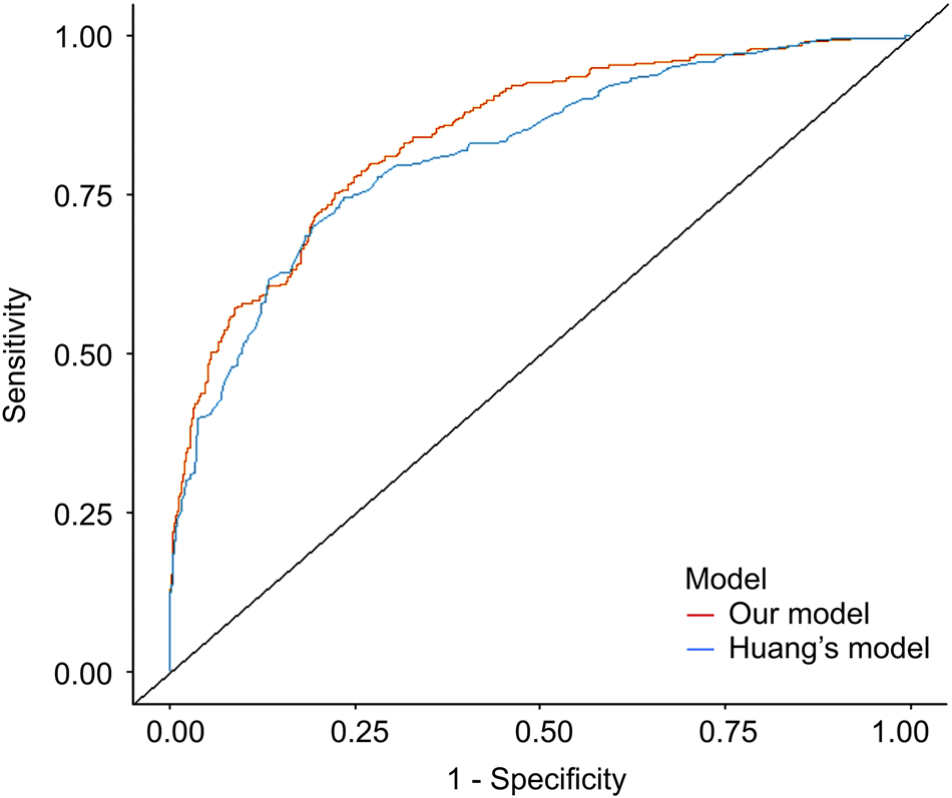
Receiver operating characteristic curve for the prediction model.

**Figure 4:**
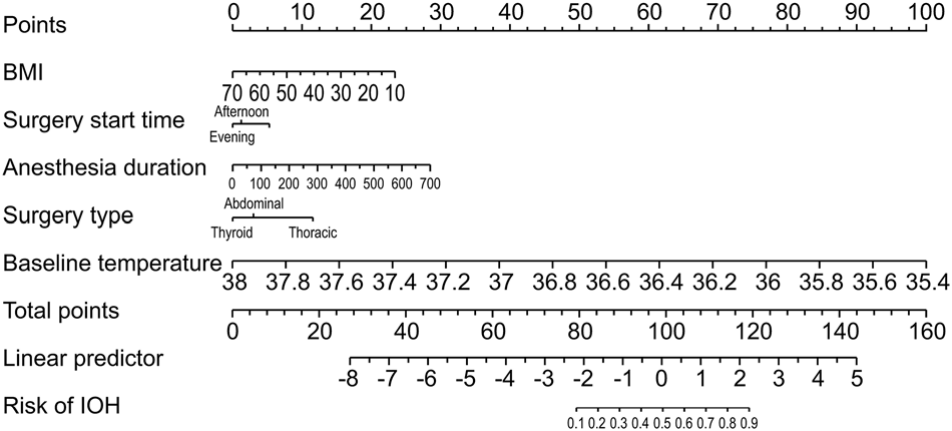
Nomogram.

Using a fivefold cross validation, we obtained a cross-validated area under the receiver operating characteristic (ROC) curve (AUROC) of 0.85 (95% CI 0.83–0.88), which indicated an excellent prediction performance. Remarkably, our model outperformed Yi J’s model (*P* = 0.001), which produced an AUROC of 0.82 (95% CI 0.79–0.85) on our data. A comparison of the two ROC curves is shown in Fig. 3. Furthermore, the nomogram for our model is shown in Fig. 4.

## Discussion

We observed an overall incidence of IOH of 41.0% in patients who underwent selective robotic surgeries. Patients with a lower BMI and baseline core temperature before anesthesia, and those who underwent morning or thoracic surgery were susceptible to hypothermia. We proposed a robotic-surgery-specific hypothermia prediction model. The model aims to identify patients with high risk for IOH in advance, such that prophylactic multimodality strategies and active warming devices can be applied to effectively prevent IOH and the associated complications. The model has demonstrated good discrimination with a cross-validated AUROC of 0.85 (95% CI 0.83–0.88).

We also observed different incidence of IOH across surgery types, which can be explained by differences in the surgical populations. We found that the incidence rates in abdominal, thoracic, and thyroid robotic surgeries were 0.41 (95% CI 0.37–0.45), 0.55 (95% CI 0.46–0.64), and 0.32 (95% CI 0.24–0.41), respectively. A survey regarding patients undergoing laparoscopic surgery in China reported that the incidence of IOH was 29.0% (200 in 690 cases). This may be due to the fact that the duration of robotic surgery tends to be longer compared with conventional laparoscopic surgery (224 vs 201 min). However, the incidence in our patients was lower than that reported in other studies, which varied from 53 to 73% in patients undergoing major abdominal and thoracic operations^12–14^. We found a higher BMI in our study (24 vs 26), probably because most bariatric surgeries are operated by robots, given their greater flexibility in narrow cavities. Consistent with other studies^1,15^, a lower BMI and baseline core temperature are important factors in developing IOH.

In this study, ambient temperature was maintained at 23 °C and an infusion warmer for fluid and blood products was mandatory in all patients, which may be a reason that our incidence was lower than that reported in previous studies in China (44.3%)^15^. The same study considered the infusion warmer as a protective factor preventing hypothermia. However, Poder et al.^16^ found that a blood warmer set at 41.5 °C is not a guarantee to avoid hypothermia when using pressure infusion cuff during massive transfusion. Because they find that the outlet temperature reached only 33.7 °C at 300 mmHg when a blood warmer set at 41.5 °C. In addition, it does not guarantee heat loss from other sources or normothermia.

Compared with anesthesia induction in the afternoon, the OR of IOH was higher in patients who were inducted in the morning. A reasonable explanation is that body temperature is generally higher in the afternoon due to circadian fluctuations in body temperature. Researchers have observed diurnal temperature variation, with a peak at 4:00 p.m.^17,18^. Other studies have shown that the core temperature is typically approximately 37.5 °C at 3:00 p.m.^19^. Additional preoperative fluid infusion for afternoon surgical cases might be another reason for hypothermia prevention.

Regarding the higher incidence of IOH in thoracic surgery, our findings are consistent with many previous studies results. Li et al.^13^ found that 72.7% (95% CI 70.5–75.0%) of adult patients underwent video-assisted thoracoscopic surgery suffered hypothermia. Emmert et al.^14^ reported an overall IOH incidence of 64.3% in thoracic surgery. Other than the less exposed skin surface for active warming in the lateral decubitus position, another possible reason is that paravertebral block is commonly combined with general anesthesia in these surgical cases, which blocks the ipsilateral sympathetic nerves and is associated with reduced thermogenesis^19^. Similarly, a previous study also confirmed that combining general anesthesia with regional anesthesia further increases the risk of IOH^9^. However, no significant difference in the incidence of hypothermia was observed between the thyroid (breast approach) and abdominal surgeries, which was probably because the exposed area was comparable according to the estimation tool for burn area known as the Lund and Browder chart^20^.

Previous studies have demonstrated that the thermoregulatory vasoconstriction threshold is reduced in elderly patients undergoing general anesthesia^14^. Except for age, another five easily accessible and confirmed by logistic regression as associated indicators, including sex, BMI, baseline core temperature, induction time, and surgical site, were further included in the prediction model for IOH. We obtained a cross-validated AUROC of 0.85 (95% CI 0.83–0.88). Notably, using the DeLong’s method, our model outperformed Huang’s formula (*P* = 0.001)^22^. Therefore, this indicates that the model has a good discriminative ability for prediction.

Our study has certain limitations. First, our analysis is single-centered, with a moderately retrospective of 833 qualified participants who underwent robotic surgery. Consequently, further extending the conclusion and prediction model is limited by sample size and specific surgery type. Second, our prediction model was influenced to some extent by unknown or potential confounders that were poorly characterized and designed in our registry (unlisted in Table 2). Systematic confounders influenced our results regarding the routine practice and device deficiency in our center, including the routine use of a warm infusion system. Few physicians chose to use these underneath warming blankets, which is a very critical issue in our experiments. However, we routinely used infusion warmers for all patients. Third, gastrointestinal, hepatobiliary, gynecology, and urology surgeries were all classified as the abdominal type of surgery, and with a sufficient sample size, these surgeries may be further analyzed under different subgroups in future studies. In addition, we excluded patients who experienced a temperature artifact lasting more than 30 min, which would introduce selection bias for patients whose temperature does not rise after treatment. Last but not the least, our model was only validated internally with the same patient population, and it awaits external validation in other institutions.

In conclusion, this study revealed a 41% incidence of IOH in patients who underwent robotic surgery. Identified risk factors showed that patients with a lower BMI and baseline core temperature and those undergoing thoracic, morning, and surgeries with longer durations were more likely to develop IOH. Our model has a good discriminative ability to predict IOH. Therefore, more effective insulation measures and the precise identification of high-risk populations are needed in clinical practice to prevent IOH and related perioperative complications.

## Methods

### Study setting and design

This retrospective single-center study enrolled patients who underwent elective robotic surgery at the China-Japan Union Hospital of Jilin University in China between June 2020 and October 2021. The informed consent was waived by the Ethics Committee and Institutional Review Board of the China-Japan Union Hospital, as the study was based on a fully deidentified database (Identifier, 20220628021). All data were obtained from institutional medical record databases.

### Data source and study sample

This study enrolled adult patients aged ≥ 18 years who were scheduled for inpatient elective robotic surgery with an expected duration of > 2 h. The exclusion criteria were as follows: (1) failed to monitor core temperature; (2) preoperative diseases affecting body temperature (e.g., hypothyroidism or hyperthyroidism, fever associated with cerebrovascular disease, high risk of malignant hyperthermia, such as medical or family history of malignant hyperthermia, and fever from infection with a core temperature higher than 38 °C before operation); (3) participation in another study within 6 months; (4) patients with a temperature artifact that lasted more than 30 min; and (5) insufficient baseline data. Furthermore, artifacts were removed using the following rules: core temperature out-of-range defined as > 38 °C or < 35 °C or abrupt changes defined by a change ≥ 0.5 °C within 5 min.

The room temperature was kept at 23 °C, while the warming blankets underneath the patients were barely used. Shortly after endotracheal intubation, the intraoperative core temperature was monitored and recorded using a disposable cord sensor (Mindray, MR410b) placed in the nasopharynx or distal esophagus. Both locations were considered reliable core temperature measurement sites^21^. Throughout the anesthesia, temperature data were automatically recorded and stored in an anesthesia recording system (Docare V5.0, Medical Systems) at 5 min intervals. Candidate factors influencing core temperature^1,9^ were collected using electronic patient record systems and nurses’ medical care records.

### Outcomes and independent variables

The primary outcome was IOH, which was defined as a core temperature of < 36 °C at any time during the perioperative procedure. The candidate influencing factors are described as follows:

1. Demographic and baseline characteristics included sex, age, BMI, American Society of Anesthesiologists physical status, and diabetes mellitus (possibly combined with impaired thermoregulation)^9^.
2. Surgery information: surgical site (thyroid, abdominal, or thoracic), blood loss, warmed or unwarmed irrigation fluid, and volume. Abdominal surgery included general, gynecological, and urological surgeries.
3. Anesthesia information included the volume of warmed intravenous fluid replacement, blood transfusion, and duration of anesthesia.
4. Other information included anesthesia induction time in the morning (8 a.m. to noon), afternoon (noon to 6 p.m.), or evening (6 p.m. to 10 p.m.); baseline core temperature, and the operating room ambient temperature.

### Statistical analyses

We first explored the changes in the intraoperative core body temperature. The body temperature trend was estimated using linear mixed models, regressing core temperature against time from anesthesia induction, using a compound-symmetric correlation matrix, and adjusting for the baseline temperature of the patients. The nonlinearity of the effect of time on the core temperature was explained using B-splines. Since the core temperature trend was changed by surgical duration, we separately plotted the curves for patients with surgical durations of 2–3 h, 3–4 h, and 4–6 h.

Although the blood loss, infusion, transfusion and irrigation are important factors for hypothermia, it cannot be accurately predicted before surgery. Therefore, we just described the data as median, first quartile (Q1), third quartile (Q3) instead of considering these factors when constructing the model.

To assess the potential risk factors for IOH in patients undergoing robotic surgery, we first summarized patient profiles by the incidence of IOH via standardized summary statistics as means ± standard deviation or n (%). Furthermore, univariate comparisons for patients with or without IOH were performed using the t-test and chi-square for continuous and categorical variables, respectively.

Subsequently, the selection of risk factors in a multivariable model was performed using backward elimination, retaining variables with *P*-values < 0.05. A fivefold cross-validated AUROC and its 95% CI were reported for the internal validation of the model. In addition, we compared our model’s predictability to that proposed by Yi et al.^22^, and the AUROC was compared using the DeLong’s method^23^. The final model, which included all patients, was reported and summarized into a nomogram.

### Sample size considerations

We estimated that approximately 45 robotic surgeries were performed monthly at our institution. A study reported an incidence of IOH of 44.3% in 3132 patients in China^16^. Assuming a more conservative incidence of 40%, we expected to collect data from 680 patients and to observe 272 IOH cases during the 16-month enrollment period. Overall, 272 IOH cases were sufficient for assessing 10 predictors, under the recommendation of 10 cases per predictor^24^.

### Ethics approval and consent to participate

All procedures were in accordance with principles of Helsinki Declaration and relevant guidelines. All protocols were approved by the Ethics Committee and Institutional Review Board of the China-Japan Union Hospital. Since it was a retrospective study based on a fully deidentified database, informed consent forms were exempted by the Ethics Committee and Institutional Review Board of the China-Japan Union Hospital.

## Supplementary Information

Supplementary Table 1.

Supplementary Figure S1.

## Data Availability

All data generated or analyzed during this study are included in this published article (and its Supplementary Information files).
