# Supplementary material for: Risk factors and prediction model for inadvertent intraoperative hypothermia in patients undergoing robotic surgery: a retrospective analysis
Source: Sci Rep. 2023 Mar 6;13:3687. doi: 10.1038/s41598-023-30819-1 (PMC9988985; doi:10.1038/s41598-023-30819-1)
Supplement: Supplementary file 1 — Supplementary Table 1. [file 41598_2023_30819_MOESM1_ESM.docx]

**Supplement table 1. Descriptive data on blood loss, transfusion, blood transfusion and irrigation fluid (N=833)**

|  | IOH (N=344) | No IOH (N=489) | *P*-value |
| --- | --- | --- | --- |
| Blood loss (ml) | 100[50, 300] | 100[50, 200] | 0.065 |
| Transfusion (ml) | 1500[1375, 2000] | 1500[1300, 2000] | 0.059 |
| Blood transfusion (ml) | 0[0, 0] | 0[0, 0] | 0.218 |
| Irrigation fluid (ml) | 400[150, 500] | 500[200, 500] | 0.273 |

IOH, inadvertent intraoperative hypothermia

Data are summarized as the median ( first quartile, third quartile [Q1, Q3])
