# Supplementary material for: Risk factors and prediction model for inadvertent intraoperative hypothermia in patients undergoing robotic surgery: a retrospective analysis
Source: Sci Rep. 2023 Mar 6;13:3687. doi: 10.1038/s41598-023-30819-1 (PMC9988985; doi:10.1038/s41598-023-30819-1)
Supplement: Supplementary file 2 — Supplementary Figure S1. [file 41598_2023_30819_MOESM2_ESM.docx]

**Supplemental Material**

**
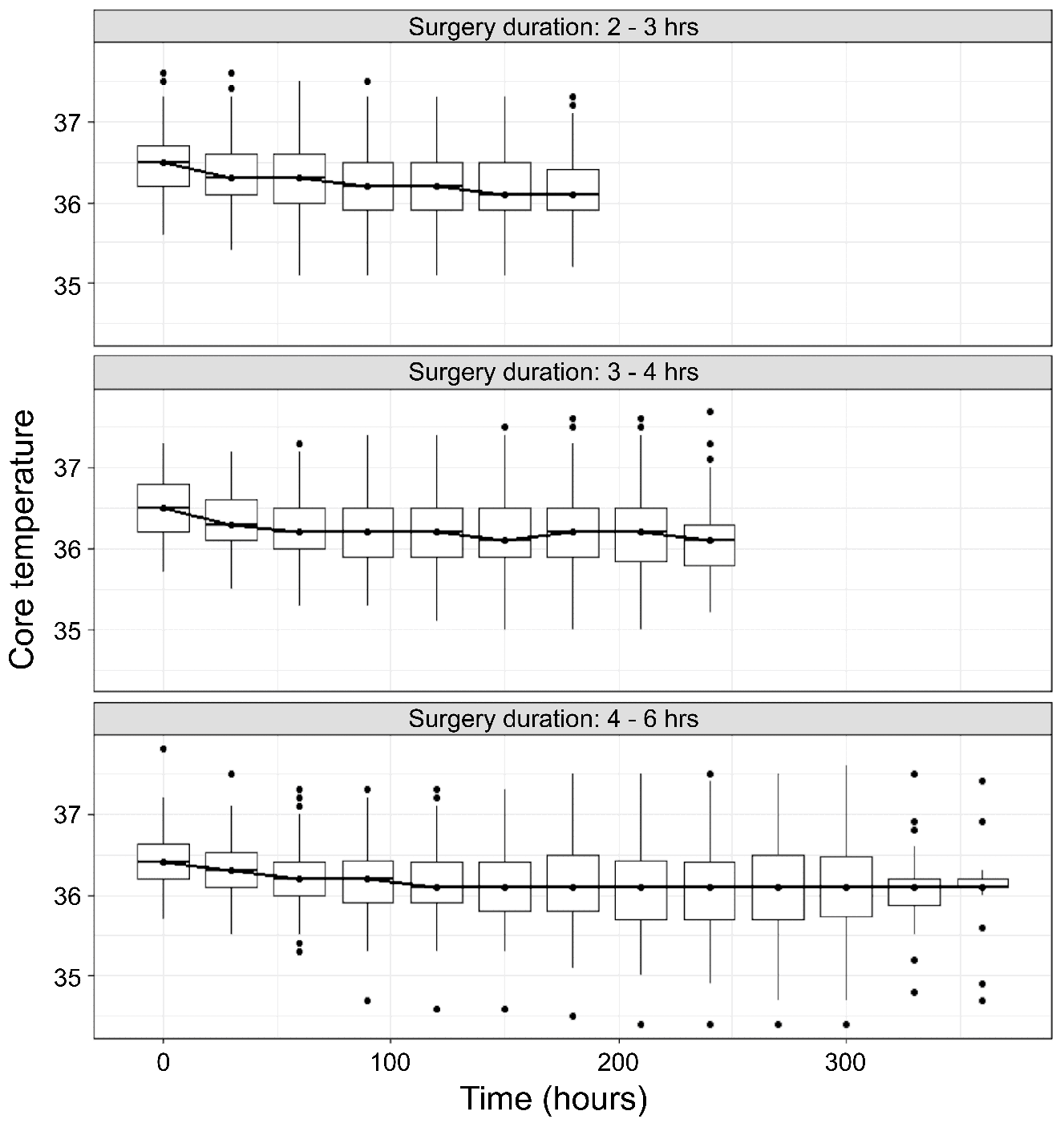
**

**Supplementary Figure S1.** Boxplot for core temperatures by surgery durations

Core temperatures were reported every 30 min, and readings lower than 30 were regarded as artifacts and were removed. Boxplots for core temperature are organized by the surgical duration of 2–3 h, 3–4 h, and 4–6 h. Since only a limited number of patients had a surgical duration of <2 h or >6 h, data from those patients were excluded from the plot.
